# Lymph node with central hypodensity

**DOI:** 10.36416/1806-3756/e20250160

**Published:** 2025-07-22

**Authors:** Edson Marchiori, Bruno Hochhegger, Gláucia Zanetti

**Affiliations:** 1. Universidade Federal do Rio de Janeiro, Rio de Janeiro (RJ) Brasil.; 2. University of Florida, Gainesville (FL) USA.

A 62-year-old male patient underwent a chest CT for staging bronchial carcinoma. The CT showed a mediastinal lymph node of normal dimensions, with central hypodensity (Figure 1).

**Figure 1:**
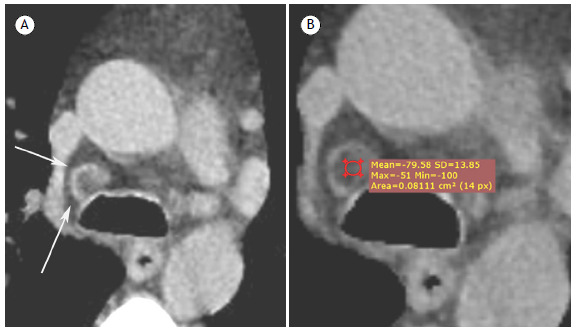
Detail of an axial CT scan of the chest, showing a mediastinal lymph node of normal dimensions (arrows), with a hypodense center (mean density = −80 HU) and a halo with greater, homogeneously thick density on the periphery.

Central hypodensity in lymph nodes may be a normal finding or correspond to a series of pathological conditions. The main parameter to be considered in terms of imaging, in addition to dimensions, is the tomographic measurement of central density. This measurement may be low, although positive, or present negative densities, generally between −30 and −150 HU, which represents the presence of fat.

Low positive densities generally indicate the presence of necrosis, both related to neoplastic processes (especially metastases) and infectious processes (tuberculosis, for example). Other less common causes are lymph node infarctions, trauma, and vasculitis.

The presence of macroscopic fat in the hilar region of lymph nodes is, in most cases, a normal finding. However, a lesser-known aspect that deserves to be highlighted is the fact that some abnormal conditions can also present with fat inside the lymph node.

This fat deposit can occur as a direct consequence of obesity, diabetes, thyroid disease, or treatment for neoplastic diseases. Generally, adipose tissue replaces the normal parenchyma, keeping the nodal volume unchanged. In some cases, the lymph nodes may reach unusual dimensions.^1^

Metastatic lymph nodes due to liposarcoma may have malignant fat cells within them. The presence of macroscopic fat expanding the lymph node may also be seen in lymphoproliferative diseases, especially in chronic lymphocytic leukemia secondary to treatment. In these cases, adipocytes in the affected lymph nodes may expand as the lymphatic tissue atrophies during treatment. Adipocytes tend to fill the void left by the atrophic process. This is important because biopsy or excision of these lymph nodes with abundant fat is usually unnecessary.^2^ ^-^ ^4^

Another interesting aspect is that the finding of lymph node adiposity on screening mammograms may serve as a useful imaging biomarker for steatotic liver disease associated with metabolic dysfunction in women at high risk for developing steatohepatitis. Early intervention in this condition may limit progression to fibrosis and end-stage liver disease.^4^

Our patient presented a lymph node of normal dimensions, with fatty content inside, surrounded by a denser halo with homogeneous thickness, fulfilling the imaging criteria of a normal lymph node.

## References

[1] Giovagnorio F, Drudi FM, Fanelli G, Flecca D, Francioso A (2005). Fatty changes as a misleading factor in the evaluation with ultrasound of superficial lymph nodes. Ultrasound Med Biol.

[2] Cooley CL, Davids MS, Giardino A (2014). Fatty intra-abdominal lymph nodes in chronic lymphocytic leukemia. Am J Hematol.

[3] Karaosmanoglu AD, Blake MA, Lennerz JK (2012). Abundant macroscopic fat in intra-abdominal lymph nodes involved in the course of a patient with chronic lymphocytic leukaemia presentation of imaging findings with biopsy correlation. Br J Radiol.

[4] Rubino JM, Ring NY, Patel K, Xia X, MacKenzie TA, diFlorio-Alexander RM (2025). Lymph Node Adiposity and Metabolic Dysfunction-Associated Steatotic Liver Disease. Biomedicines.

